# Anxiety and depression in pregnant women who have experienced a previous perinatal loss: a case-cohort study from Scandinavia

**DOI:** 10.1186/s12884-022-05318-2

**Published:** 2023-02-13

**Authors:** Anustha Mainali, Jennifer J. Infanti, Suraj Bahadur Thapa, Geir W. Jacobsen, Tricia L. Larose

**Affiliations:** 1 Department of Community Medicine and Global Health, Institute of Health and Society, Faculty of Medicine, University of Oslo, Oslo, Norway; 2 Department of Public Health and Nursing, Faculty of Medicine and Health Sciences, Norwegian University of Science and Technology, Trondheim, Norway; 3 Division of Mental Health and Addiction, Institute of Clinical Medicine, Faculty of Medicine, University of Oslo, Oslo, Norway; 4 Present Address: Department of Clinical and Molecular Medicine, Faculty of Medicine and Health Sciences, Norwegian University of Science and Technology, Trondheim, Norway

**Keywords:** Perinatal loss, Subsequent pregnancy, Anxiety, Depression, Maternal mental health, Scandinavian successive small-for-gestational age births study (SGA study)

## Abstract

**Background:**

Perinatal loss can have long-lasting adverse effects on a woman’s psychosocial health, including during subsequent pregnancies. However, maternal mental health status after perinatal loss during subsequent pregnancy is understudied with very little data available for Scandinavian populations.

**Aims:**

The primary aim of the study was to explore the association between previous perinatal loss and anxiety/depression symptoms of expectant mothers during the subsequent pregnancy. The secondary aim of this study was to explore possible determinants of maternal mental health during the subsequent pregnancy, independent of previous perinatal loss.

**Method:**

This case-cohort study is based on primary data from Scandinavian Successive Small-for-Gestational Age Births Study (SGA Study) in Norway and Sweden. The total case-cohort sample in the current study includes 1458 women. Cases include 401 women who had reported a previous perinatal loss (spontaneous abortion, stillbirth, or neonatal death) and who responded to two mental health assessment instruments, the State-Trait Anxiety Inventory (STAI), and the Centre for Epidemiological Studies Depression (CES-D) scale. Multiple linear regression models were used to assess the association between previous perinatal loss and maternal mental health in subsequent pregnancy.

**Results:**

Scandinavian pregnant women with previous perinatal loss reported higher symptoms for both anxiety and depression during their subsequent pregnancy compared to mothers in the same cohort reported no previous perinatal loss. Multiple linear regression analyses showed a positive association between previous perinatal loss and per unit increase in both total anxiety score (β: 1.22, 95% CI: 0.49–1.95) and total depression score (β: 0.90, 95% CI: 0.06–1.74). We identified several factors associated with maternal mental health during pregnancy independent of perinatal loss, including unintended pregnancy despite 97% of our population being married/cohabitating.

**Conclusion:**

Women who have experienced previous perinatal loss face a significantly higher risk of anxiety and depression symptoms in their subsequent pregnancy.

## Background

The World Health Organization (WHO) defines perinatal loss as the death of a fetus or baby/neonate between 20 completed weeks of pregnancy and up to 7 days after birth [1]. Globally, 5.1 to 5.3 million stillbirths and neonatal deaths occurs annually [2]. Twenty-three million miscarriages occur every year accounting for a global stillbirth rate of 13.9 per 1000 total births [3, 4]. As of 2015, perinatal deaths contributed to 50% of under-5 mortality globally [5]. Although the majority of these events occur in low-and-middle-income countries [2], the profound personal, family and societal impact of perinatal loss is felt around the globe, including in high-income countries [6]. In the European context, perinatal mortality has declined over the last 30 years with a similar trend in Nordic countries. In 2019, the perinatal mortality rate amongst all member countries of the European Union was 5.5 deaths per 1000 live and stillbirths, and in Nordic countries 2.6 deaths per 1000 live and stillbirths [7]. More specifically, in 2020 the perinatal mortality rate in Norway was 3.9 per 1000 live and stillbirths and 1.4 per 1000 live and stillbirths in Sweden [5, 8, 9]. In Norway, this translates to approximately 200 stillbirths each year [10] with no identified reasons or causes [9].

Previous perinatal loss has the potential to cause poor mental health sequela among women during subsequent pregnancy, including prolonged and complicated grief, anxiety, depression, posttraumatic stress, marital disruption, and suicidal ideation [11]. An experience of perinatal loss can have long-lasting adverse effects on a woman’s psychosocial health, including during subsequent pregnancies [12, 13]. The phenomenon of maternal mental health status in a subsequent pregnancy amongst women who have experienced a previous perinatal loss is better studies in the North America [14–16] and some European countries [17, 18] . Unfortunately, there is a paucity of evidence for Scandinavia.

A meta-analysis by Hunter et al. provided estimated rates of anxiety, depression and stress in 5114 pregnant women (age range 15–46 years) and 30,272 controls during pregnancies after previous perinatal loss over a 20-year period [19]. Only one Scandinavian study [20] was cited in this meta-analysis to provide evidence on the value of long term follow-up to help reduce higher rates of mortality in mothers with previous perinatal loss, but this single Scandinavian study was not used for modelling purposes since the end-point was mortality and not mental health. We did find one key Norwegian study [21] in the literature with a smaller sample size than the current study (901 pregnant women) and different methodology (cohort linked to registry). Data from the Norwegian Mother, Father and Child Cohort Study (MoBa) [21], a population-based pregnancy cohort, showed that Norwegian pregnant women after stillbirth had a higher prevalence of anxiety (22.5%) and depression (19.7%) compared to Norwegian pregnant women with a previous live birth, or previously nulliparous women. As far as we know, Swedish data on this topic is not readily available in the English language scientific literature.

The primary aim of the present study was to explore the association between previous perinatal loss and anxiety/depression symptoms of expectant Norwegian and Swedish mothers during their subsequent pregnancy. The secondary aim of this study was to explore possible determinants of maternal mental health during the subsequent pregnancy, independent of previous perinatal loss.

## Methods

This study used a case-cohort design based on data from the Scandinavian Successive Small-for-Gestational Age Births Study (SGA Study) in Norway and Sweden. This SGA study was conducted in joint collaboration by three Scandinavian universities of Trondheim, Bergen (Norway) and Uppsala (Sweden) with the U.S. Epidemiology and Biometry Research Program at the National Institute of Child Health and Human Development (NICHD) [22, 23]. Recruitment of multigravida pregnant women was conducted at the university hospitals in Trondheim and Bergen (Norway) and Uppsala (Sweden). Based on this recruitment, a mother-child cohort (1986–1988) in Norway and Sweden was established including long-term follow-up of mother and child [22]. The SGA study includes comprehensive data collected from interviews, standardized questionnaires, self-study forms, and clinical examinations at 17, 25, 33 and 37 weeks of gestation, and at birth [22].

The total case-cohort sample in the current study includes 1458 women from the original 2072 women. Cases include 401 women who had reported a previous perinatal loss (spontaneous abortion, stillbirth, or neonatal death) and who responded to two mental health assessment instruments, the State-Trait Anxiety Inventory (STAI), and the Centre for Epidemiological Studies Depression (CES-D) scale. This took place at the antenatal care visit during gestational week 25 of their subsequent (index) pregnancy. Non-cases included 1057 women who did not report previous perinatal loss but responded to the same mental health instruments, STAI and CES-D, at the same antenatal visit during their subsequent pregnancy.

### Definition of previous perinatal loss

The current study defined a previous perinatal loss according to the WHO [1] as the previous death of a fetus or neonate between 20 completed weeks of pregnancy and up to 7 days after birth, including previous stillbirth or neonatal death. Confirmation of previous perinatal loss among SGA study participants was recorded at first study visit of subsequent pregnancy (gestational week 17).

### Measurements of maternal mental health

Maternal mental health status (anxiety and depression) during a subsequent pregnancy after a perinatal loss was assessed at gestational week 25 by use of two validated instruments: State-Trait Anxiety Inventory (STAI-X) [24], and CES-D for depression [25].

#### Anxiety

Maternal anxiety was measured using the STAI-X trait anxiety scale, a structured mental health screening instrument designed for the assessment of the intensity of feelings [24]. It consists of two questionnaires, each of 20 items, one for state anxiety and the other for trait anxiety. The STAI-X (trait version) to measure the disposition of pregnant women towards their feelings of anxiousness and worthlessness [24]. Each of the items is rated on a 4-point scale, asking the pregnant women to evaluate how she generally feels (trait anxiety). Thirteen questions in the tool are positively scored (0 = 0, 1 = 1, 2 = 2 and 3 = 3) and 7 questions (1, 6, 7, 10, 13, 14 and 16) are reversely scored (0 = 3, 1 = 2, 2 = 1 and 3 = 0). The STAI-X total score ranges from 20 to 80 [24, 26]. There are no standard cut-off scores established for this inventory, but previous studies have used raw cutoff scores of > 45 as an indication of high trait-anxiety [26, 27].

#### Depression

The CES-D is a short, structured, self-report 20 item screening tool for depression [17]. It is a quick self-test that measures depressive feelings and behaviors in four domains: depressed affect, positive affect, somatic activity, and interpersonal relations over the past week [25, 28]. Participants are asked to indicate how many days in the previous week they experienced depressive symptoms. The response options are rated on a four-point Likert scale that range from 0 to 3 for each item (0 = rarely or < 1 day, 1 = sometimes or 1–2 days, 2 = occasionally/moderately or 3–4 days, 3 = mostly or 5–7 days). Total scores (range 0–60) reflect the level of depression experienced by the participant over the last week. A respondent with a total score of < 16 is interpreted as at “no risk” of depression, whereas a total score of 16–21 indicates mild to moderate depression, and a total score ≥ 22 represents severe depressive symptoms [28]. Sixteen questions in the tool are positively scored as opposed to four questions (4, 8, 12 and 16) which are reversely scored. Reliability and validity of this tool are well established, and the instrument has been widely used in epidemiologic studies [29]. The CES-D scale has been widely used to assess depressive symptomatology during pregnancy [30, 31] and has good psychometric properties [17]. It is comparatively different from other depression scales since it is not designed for clinical diagnosis but is based on symptoms of depression seen in clinical cases [25].

### Potential factors associated with maternal mental health

Several factors that may affect maternal mental health status independent of previous perinatal loss were chosen based on a priori evidence from the literature and their known association with anxiety and/or depression symptoms amongst pregnant women. These factors included maternal age (at time of study entry, continuous), civil status (married/cohabitating or single), maternal education (elementary school, high school or college/university), occupational status (full time, part time or no salaried word), woman’s own description of family’s current economic situation (good, medium or bad), ability of a woman to raise 5000 Norwegian kroner (NOK) in one week (yes or no) as an indication of ability to raise a quasi-substantial sum of money within a 1-week time frame from a bank, friend, family member, maternal smoking during pregnancy (yes or no). According to Norges Bank, 5000 NOK in 1988 is equal to 10,5777 NOK in 2021 [32]. This amount could be equivalent to monthly rental housing fee for a one or two bedroom apartment, depending on the geographic location. Maternal alcohol consumption during pregnancy (never, < 1/month or ≥ 1/month), whether or not the pregnancy was planned (yes or no) and history of mental illness (yes or no). Data on these variables were collected at study intake (week 17), except for alcohol consumption and pregnancy intention that were collected at gestational week 33.

### Statistical methods

Descriptive summary statistics are presented as mean, standard deviation (SD) for continuous variables and proportions for categorical variables for the total case-cohort population (*N* = 1458), and for cases (*n* = 401) and non-cases (*n* = 1057) separately. Multiple linear regression models were used to assess the association between previous perinatal loss and maternal mental health in subsequent pregnancy. Total anxiety score and total depression score were considered continuous outcome variables and were run as two separate models before and after adjustment for potential confounders. Simple linear regression was used to explore potential factors that may be associated with maternal mental health – independent of previous perinatal loss. Results from the linear regression models are presented as beta coefficient (β) and 95% confidence intervals (95% CI). Preliminary analysis was conducted to ensure no violation of the assumption of normality, linearity, and multicollinearity. Statistical analyses were performed using SPSS Version 28 [33].

## Ethics

All methods in this study were carried out in accordance with relevant guidelines and regulations. Research was performed in accordance with the Declaration of Helsinki. Informed consent was obtained by all SGA study participants and/or their legal guardians. This study was approved by the Regional Ethics Committee, Uppsala, Sweden (Dnr 2015/067). As of 01 January 2021, ongoing analysis of the SGA Study data is based solely on anonymous data. Based on this anonymity, the Norwegian Center for Data Research has decided that no further of assessment of data usage for the SGA study is necessary.

## Results

A total of 1458 women (401 cases and 1057 non-cases) were included in the current study. The baseline characteristics between cases and non-cases were quite similar (Table 1). Study participants were on average between 28 and 29 years old. The vast majority of women were married or cohabitating (97%) and had a good or medium family economic situation (< 90%). Still, rather few women were able to raise NOK 5000 in one week (11%). Nearly three-quarters of the study participants reported maternal smoking during pregnancy which was expected since the SGA study population is enriched with current smoking as a risk factor for small for gestational age birth. Less than one in ten women reported any alcohol consumption during the second part of pregnancy. The pregnancy was planned for three-quarters of the study population, regardless of case vs. non-case status, and very few women reported previous mental health problems (< 2%).

**Table 1:** Characteristics of Study Population

Variables	Total (***N*** = 1458)	Cases (401)	Non- Cases (1057)
**Maternal Age (Mean, SD)** ^**1**^	28.48 (4.2)	29.39 (4.5)	28.11 (4.1)
**Civil status (n, %)**			
Married/Cohabiting	1420 (97.4)	391 (97.5)	1029 (97.4)
Single	33 (2.3)	9 (2.2)	24 (2.3)
Missing	5 (0.3)	1 (0.2)	4 (0.4)
**Education (n, %)**			
Elementary School	288 (19.8)	79 (19.7)	209 (19.8)
High School	755 (51.8)	211 (52.6)	544 (51.5)
College/University Degree	407 (27.9)	110 (27.4)	297 (28.1)
Missing	8 (0.5)	1 (0.2)	7 (0.7)
**Occupational status (n, %)**			
Full time	418 (28.7)	126 (31.4)	292 (27.6)
Part time	614 (42.1)	152 (37.9)	462 (43.7)
Non-salaried work	406 (27.8)	117 (29.2)	289 (27.3)
Missing	20 (1.4)	6 (1.5)	14 (1.3)
**Economic situation, (n, %)**			
Good	688 (47.2)	194 (48.4)	494 (46.7)
Medium	657 (45.1)	172 (42.9)	485 (45.9)
Bad	80 (5.5)	27 (6.7)	53 (5.0)
Missing	33 (2.3)	8 (2.0)	25 (2.4)
**Able to raise NOK 5000 in a week, (n, %)**			
Yes	161 (11.0)	48 (12.0)	113 (10.7)
No	1262 (86.6)	343 (85.5)	919 (86.9)
Missing	35 (2.4)	10 (2.5)	25 (2.4)
**Smoking status (n, %)**			
Yes	1080 (74.1)	297 (74.1)	783 (74.1)
No	376 (25.8)	103 (25.7)	273 (25.8)
Missing	2 (0.1)	1 (0.2)	1 (0.1)
**Alcohol consumption (n, %)**			
Never	682 (46.8)	206 (51.4)	476 (45.0)
Not as often as once a month	485 (33.3)	111 (27.7)	374 (35.4)
> Once a month	238 (16.3)	68 (17.0)	170 (16.1)
Missing	53 (3.6)	16 (4.0)	37 (3.5)
**Planned pregnancy (n, %)**			
Yes	1082 (74.2)	310 (77.3)	772 (73.0)
No	319 (21.9)	76 (19.0)	243 (23.0)
Missing	57 (3.9)	15 (3.7)	42 (4.0)
**Previous history of mental health problem (n, %)**			
Yes	21 (1.4)	6 (1.5)	15 (1.4)
No	1425 (97.7)	394 (98.3)	1031 (97.5)
Missing	12 (0.8)	1 (0.2)	11 (1.0)

^1^SD: Standard Deviation

The mean depression score for cases compared to non-cases was slightly higher (9.5 ± 7.4 vs. 8.6 ± 7.2, respectively) with a larger proportion of cases compared to non-cases (19% compared to 15%, respectively) scoring ≥16 on the CES-D (data not shown).

Linear regression analysis showed a positive association between previous perinatal loss and per unit increase in both total depression score (β: 0.92, 95% CI: 0.80–1.75) and total anxiety score (β: 1.27, 95% CI: 0.55–2.10) (Table 2). These associations persisted even after adjustment for maternal age, civil status, education, occupation, economic situation of family, ability to raise NOK 5000 in one-week, smoking status, alcohol consumption, pregnancy intention and previous history of mental health: anxiety (β: 1.22, 95% CI: 0.49–1.95), depression (β: 0.90, 95% CI: 0.06–1.74).

**Table 2:** Regression coefficients for depression and anxiety score by previous perinatal loss

Previous Perinatal Loss	Model 1: Unadjusted		Model 2^**a**^: Adjusted	
	Depression (β, 95% CI)	Anxiety (β, 95% CI)	Depression (β, 95% CI)	Anxiety (β, 95% CI)
**Yes**	0.92 (0.80, 1.75)	1.27 (0.55, 2.1)	0.90 (0.06, 1.74)	1.22 (0.49, 1.95)
**No**	Reference	Reference	Reference	Reference

^a^ Adjusted for maternal age at study entry, civil status, education, occupation, economic situation of family, ability to raise 5000 NOK in 1 week, alcohol consumption, smoking, pregnancy intention and previous history of mental health problems

Smoking during pregnancy and unintended pregnancy were associated with both increased total anxiety score and increased total depression score, whereas good family economy was negatively associated with anxiety and depression scores (Table 3). Several additional factors were associated with depression but not with anxiety. No salaried work was associated with an increase in total depression score (compared to working full time), and being married/cohabitated, as well as having higher education was associated with a per unit decrease in total depression score.

**Table 3:** Univariate linear regression analysis for potential factors associated with depression and anxiety symptoms

Variables	Depression				Anxiety			
	β	SE	95% CI	***P***	β	SE	95% CI	***P***
**Maternal age**	− 0.03	0.05	(− 0.12, 0.06)	0.570	0.07	0.04	(−0.01, 0.14)	0.093
**Civil Status**								
Single	4.13	1.28	(1.63, 6.63)	0.001	0.65	1.10	(−1.52, 2.81)	0.560
Married/Cohabiting	Ref	Ref	Ref	Ref	Ref	Ref	Ref	Ref
**Education**								
Elementary school	1.65	0.56	(0.55–2.75)	0.003	0.36	0.48	(−0.58, 1.31)	0.450
High School	0.75	0.46	(− 0.13, 1.62)	0.095	− 0.22	0.39	(− 0.98, 0.54)	0.571
College/University	Ref	Ref	Ref	Ref	Ref	Ref	Ref	0.000
**Occupational status**								
Non-salaried work	1.77	0.51	(0.78, 2.76)	< 0.001	0.64	0.44	(−0.21, 1.50)	0.141
Part time	−0.04	0.46	(−0.94, 0.86)	< 0.93	− 0.47	0.40	(−1.25, 0.31)	0.30
Full time	Ref	Ref	Ref	Ref	Ref	Ref	Ref	Ref
**Economic Situation**								
Bad	4.19	0.85	(2.53, 5.85)	< 0.001	2.67	0.73	(1.24, 4.10)	< 0.001
Medium	0.65	0.39	(0.12, 1.42)	0.097	0.68	0.34	(0.02, 1.34)	0.044
Good	Ref	Ref	Ref	Ref	Ref	Ref	Ref	Ref
**Ability to raise NOK 5000 in a week**								
No	−2.82	0.60	(−3.10, −1.64)	< 0.001	−1.43	0.52	(−2.45, −0.41)	0.006
Yes	Ref	Ref	Ref	Ref	Ref	Ref	Ref	Ref
**Smoking status**								
Yes	2.24	0.43	(1.40, 3.09)	< 0.001	1.34	0.37	(0.60, 2.07)	< 0.001
No	Ref	Ref	Ref	Ref	Ref	Ref	Ref	Ref
**Alcohol consumption**								
>Once a month	0.02	0.55	(−1.06, 1.09)	0.975	0.75	0.47	(−0.17, 1.67)	0.111
Not as often as once	−0.10	0.43	(−0.95, 0.74)	0.814	0.51	0.37	(−0.22, 1.24)	0.173
Never	Ref	Ref	Ref	Ref	Ref	Ref	Ref	Ref
**Planned pregnancy**								
No	2.45	0.46	(1.56, 3.35)	< 0.001	1.39	0.47	(0.61–2.17)	< 0.001
Yes	Ref	Ref	Ref	Ref	Ref	Ref	Ref	Ref
**Previous mental health problem**								
Yes	−0.17	1.59	(−3.29. 2.95)	0.916	0.57	1.37	(−2.12, 3.26)	0.678
No	Ref	Ref	Ref	Ref	Ref	Ref	Ref	Ref

## Discussion

The current case-cohort study showed that Norwegian and Swedish mothers who experienced previous perinatal loss reported higher symptoms for both anxiety and depression in their subsequent pregnancy compared to mothers in the same cohort who had no such experience of previous perinatal loss.

Our results concur with at least one Scandinavian study from the literature [21]. This study linked data from the MoBA birth cohort and data on stillbirth from the Medical Birth Registry of Norway – a registry based on compulsory notification of all live births, stillbirths and late miscarriages or terminations of pregnancy in Norway [34]. However, only 41% (*n* = 901) of invited mothers from MoBa agreed to participate (174 pregnant after a stillbirth, 362 pregnant after a live birth and 365 previously nulliparous). Results indicate that Norwegian pregnant women after stillbirth have a higher prevalence of anxiety (22.5%) and depression (19.7%) compared to Norwegian pregnant women with a previous live birth, or previously nulliparous women. There are important differences between this MoBa study and the current study. First, the current study includes both Norwegian and Swedish mothers with a larger sample size, and the current study recruited women between 1986 and 1988 whereas the MoBa study included participant data from 1999 to 2008. Our data on stillbirths were self-reported during clinical exam at study entry whereas the MoBa study leveraged registry based data. The end-points between these two studies are not directly comparable since different statistical methods applied to different psychometric tests were used. However, both studies conducted robust analyses with ample sample sizes on using validated and standardized assessment tools. Taken together, results from the MoBa study and results from the current study validate the important finding that Norwegian and Swedish women who have experienced a perinatal loss are at increased risk of both anxiety and depression during subsequent pregnancy compared to women who have not experienced a perinatal loss.

As mentioned, evidence from North America and Europe is more readily available than evidence from Scandinavia. For example, a recent study from Germany [19] also supports our findings. The authors conducted an assessment of 155 pregnant women and collected repeated measures of maternal stress and mood, on average eight times per day over a consecutive 4-days period. Women with a history of prenatal loss (*n* = 40) reported higher levels of pregnancy-specific distress in early as well as late pregnancy. These women were more nervous and tired compared to other pregnant women. In the comparison group, pregnancy-specific distress decreased, and mood improved from early to late pregnancy. No evidence of decreased distress or improved mood throughout pregnancy was observed amongst pregnant women who experienced a previous perinatal death.

The mental health impact of pregnancy loss in general is better understood than the mental health impact of pregnancy loss during the subsequent pregnancy or after a subsequent live birth. For example, a 2022 systematic review and meta-analysis [35] by Herbert et al. identified 29 studies from 17 countries with 31,072 women who experienced a perinatal loss and more than 1.2 million controls. Results from their random-effects modelling suggest women who experienced a perinatal loss were at increased risk of anxiety and depressive disorders, and increased anxiety/depression scores compared to women who experienced no such loss. With such robust evidence on hand, assessing maternal mental health following perinatal loss until and throughout subsequent pregnancy ought to be a maternal health priority.

Our evidence, supported by one previous study in Norway and findings from other non-Scandinavian populations, clearly suggest that the grief trajectory of perinatal loss extends into subsequent pregnancies. Additional evidence suggests that the grief trajectory of perinatal loss, including symptoms of anxiety and depression, can extend beyond the subsequent pregnancy to 6-months after a healthy live birth [16], and up to 134-months postpartum [36].

Whilst previous perinatal loss is a clear determinant of anxiety and depression during subsequent pregnancy, other factors independent of perinatal loss or interacting with perinatal loss can also impact maternal mental health during pregnancy. In the current study we found several factors to be associated with maternal mental health when considering the cohort as a whole. Unintended pregnancy was associated with increased anxiety and depression symptoms in our study despite the vast majority of married or cohabitating women in our population (97%). This seems to suggest that it is intentionality in and of itself that is a predictor for anxiety and depression among pregnant women, as opposed to unintended pregnancies among women who may lack social support. For example, Fellenzer et al. 2014 [37] showed that women with unintended pregnancies were more likely (AOR, 95% CI) to report severe (3.6, 2.6–5.1) or moderate (2.0, 1.6–2.5) prenatal depression symptoms and less likely to report no symptoms, compared to women with intended pregnancies. However, nearly half of the women in this study were unmarried, and unmarried status was also found to be a predictor of anxiety and depression. Our finding of intentionality despite marital status needs confirmation in future studies.

Smoking during pregnancy was also associated with both increased total anxiety score and increased total depression score in our study. However, the SGA Study, upon which this current case-cohort study is based, is enriched with smokers since smoking is a risk factor for small-for-gestational age births. We therefore assume that we have over-estimated the association between maternal smoking and maternal mental health during pregnancy and suggest that this finding be considered with caution. Still, this finding is consistent with a large body of evidence which shows substance use by pregnant women during the perinatal period is associated with a variety of negative sequelae that could impact the mother, pregnancy outcomes, and the child in a lifetime perspective [38].

Several additional factors were associated with depression but not anxiety in the current study. Pregnant mothers who reported no salaried work had an increase in total depression scores compared to pregnant mothers who were working full time. This is unsurprising considering unemployment/poor socioeconomic status is a known marker for poor mental health leading to anxiety and depression, in general [39]. More specifically, lower socioeconomic position (occupation, ability to raise money, and family’s current economic situation) has been directly associated with the burden on maternal mental health [40, 41]. In line with this previous research, our study also showed that women who self-reported their family economy as good (compared to medium or bad) had lower total anxiety and lower total depression scores. We also show that a woman’s ability to raise NOK 5000 in one week was protective against anxiety, and higher education was associated with a per unit decrease in total depression score. It is interesting to note that socioeconomic position continues to play a role as a determinant of health even within countries that have a strong social welfare state.

This study has several strengths including a large sample size, and use of standardized psychometric instruments to measure anxiety (STAI-X) and depression (CES-D). Our cohort was homogeneous, including multigravida cases and non-cases, and we controlled for a number of potentially confounding factors. At the same time, the homogeneity of our populations may limit the generalizability of our findings, and the data leverage for this study is historical. Still, this study fills a significant gap in the literature with particular relevance to Scandinavian countries and will serve as baseline data to future longitudinal studies in the same cohort that includes data from mother-child pairs at 26- to 28-year follow-up. We did not have data on the index perinatal loss. The STAI-X tool used in the current study was missing two questions for all study participants. These missing questions/responses impact the total anxiety scores for all women in our study population. However, our use of linear regression with total anxiety score as a continuous outcome minimizes potential impact of these missing questions.

## Conclusions

In this large case-cohort study of multigravida pregnant women from Norway and Sweden, we found clear associations between previous perinatal loss and increased total anxiety and total depression scores. We also identified several factors associated with maternal mental health independent of previous perinatal loss. Given the adverse outcomes and long-term grief trajectory of previous perinatal loss on maternal mental health, preventive interventions are needed to reduce the burden of illness, provide coping strategies, and promote healthy adjustment of pregnant women in a life-course perspective.

## Data Availability

The datasets used and/or analysed during the current study may be made available from the corresponding author on reasonable request.

## Abbreviations

AOR: Adjusted Odds Ratio
CES-D: Centre for Epidemiological Studies Depression Scale
CI: Confidence Interval
SD: Standard Deviation
MoBa: The Norwegian Mother and Child Cohort study
NICHD: National Institute of Child Health and Human Development
SGA Study: Scandinavian Successive Small-for-Gestational Age Study
STAI: X State-Trait Anxiety Inventory
WHO: World Health Organization
